# Supplementary material for: Cessation of breastfeeding in mothers of preterm infants—A mixed method study
Source: PLoS One. 2020 May 15;15(5):e0233181. doi: 10.1371/journal.pone.0233181 (PMC7228110; doi:10.1371/journal.pone.0233181)
Supplement: Attachment — Submitted filename: Reviewers report Manuscript Number PONE-D-20-00937.docx [file pone.0233181.s001.docx]

**Manuscript Number PONE-D-20-00937: Cessation of breastfeeding in mothers of preterm -a mixed method study**

The study aimed at describing maternal and infant behaviors accounting for cessation of breastfeeding in mothers of preterm infants up to 12months after birth. The study employed a mixed method design using a convergent approach.

**Comments to the Author**

The manuscript could be published following major revisions. Well done.

**Title:** It is clear and concise.

**Abstract**: Abstract was stated clearly.

Line 40: You need to include the method of data analysis in the methods section of the abstract.

Could include the themes identified in the results section of the abstract.

**Introduction:** The introduction was supported by relevant literature however there is a need to provide references for statements made in lines 58, 64-65.

**Material and methods**: The design is appropriate for the study. Though it was stated that 270 mothers were used for the study in the abstract as well as the results section of the manuscript, the inclusion criteria were not explicitly stated in relation to both mother and infant characteristics. For example, category of preterm infants - extremely, preterm, very preterm or moderate to late preterm. The sampling technique was also not stated. Measures used to ensure validity and reliability of the items in data collection instrument for the quantitative part of the study as well as those used to ensure trustworthiness in the qualitative part should be included.

Line 87-89: The statement is not necessary.

Line 90-91: Please revise the sentence.

Line 94-95: Instead of “breastfeeding period” please revise it to read “the first year of life”.

Line 99: It will be appropriate to define the terms exclusive breastfeeding and partial breastfeeding as used in the study.

Line 102: How was the breastfeeding satisfaction analyzed using the visual analogue scale?

Line 105-106, 111-112: What is the difference between the two questions stated?

Line 111: Please clarify which set of questions were asked at the different points.

**Results**

Line 117-118: Please revise this sentence for clarity.

Line 124: Was this the only condition for conducting a Mann -Whitney U test analyses? You need to assess the data to see if they meet the assumptions for parametric testing or not before selecting the appropriate statistical test.

Line 125: Chi-square test is used to measure association between two categorical variables and not for measurement of differences.

Line 129: Only one open ended question (line 109-110), has been stated in the manuscript. What was the second open ended question?

Line 134-137: There is a need to clearly define each of the alphabets or letters being used to describe participants and breastfeeding cessation for readers to understand.

Line 138-140: Please revise the statement for clarity.

Line 149-150: For the written comments about breastfeeding cessation did participants provide more than one specific comment? (165 comments from 149 participants)

Table 1. The numbers in the table do not add up to the total number of participants

What was response rate in terms of the questionnaire sent out. Where the questionnaires self-administered?

Table 1: “Partial breastfeeding 12 months after birth”. This percentage seems inaccurate, what was the denominator used for this calculation?

Table 2: Low maternal educational level…These numbers in the brackets do not sum up to 100%. What does this mean? If the numbers that responded to each question item are not same, you can put the total numbers beside the item.

Were multiparas involved in this study? If yes, were there any differences found in breastfeeding cessation between primiparas and multiparas?

If different categories of preterm infants were involved in the study, were there any differences in terms of breastfeeding cessation?

Line 172: Two open ended questions meanwhile there is only one in the manuscript.

Line 174: Please reframe this sentence to clarify the main theme.

Line 179: “Negative feedback”. What exactly does this mean?

Line 182-183: If participant stopped breastfeeding at 3 months why the use of letter ‘p’ at 3 months. Please clarify.

Line: 185 -186: Please move this statement upwards, i.e., after the first two statements and before the statement beginning with "From the mothers’ descriptions ..." (line 177)

Line 189: “There was a difference in this theme” This is unclear, please revise

Line 219: Please be specific, “this pain was because the infant teeth came in”. Do you mean the infants with teeth were biting on the breast?

Line 220- 221: “In addition, the infant health such as cowmilk protein allergy…” This statement seems unclear please consider revising.

Line 224: If participant ceased breastfeeding at 8 months why is the same participant categorized as practicing exclusive breastfeeding at 12 months?

Line 246: Why was the quote of the participant referenced as [16]?

Line 260: Mothers who did not breastfeed as long as they wanted were “less active …….” Did you observe this or this is an information they told you? Please clarify.

Line 248- 250: The sentence looks incomplete.

Line 263 What is the meaning of “harmonious breastfeeding”?

Line 264: How powerful can these words be when they are describing their experience?

Line 266: ……. “of pre-determined biologically given end “this section seems unclear, please revise.

Line 270: What does it mean for the mother to be “unreflective in relation to her own body”?

**Discussion**

Line 306: You need to cite more than one reference as you made mention of “studies”

Line 307: Same comment as line 306 above

Line 314: “The mothers did not seem to do anything active about their milk supply” What does this mean and what were the expectations in such situations?

Line 318: “Cessation of breastfeeding is a fact” revise this phrase.

Line 332- 333: “……the cultural influence and that mothers seem……” This information was not reflected in your results.

Line 334: In what ways can mothers be strengthened?

Line 343: Approximation of 35 weeks of breastfeeding to 12 months needs to be clarified.

Line 345: ….. “when it was cultural acceptable to cease breastfeeding, this information did not reflect in your results.

Line 346: To what extent is breastfeeding for 2-4 years in non-industrial populations comparable to Sweden? Please expand the point for clarity especially with respect to the early cessation of breastfeeding reported in Sweden.

Line 348-349: Hypothesis? Please provide references for the statements made.

Line 354: “A limitation is that comments left by mothers were relatively short”. If this is addressed as a limitation, how does the qualitative data adds up to the strength of your study as earlier described in line 352?

Line 355: “width and depth”, What does this mean in the context of your study?

**Conclusion:** The conclusion was concise and the implications for practice was stated.

**Limitations**: The limitations of the study were emphasized.

**References:** All references used were accounted for in the list of references, however there is a need to cross check reference number [16] since it was used as in text citation for a quote by a participant (Line 246).
